# TREM2‐Mediated Myeloid Cells Protect Against Pathological Choroidal Neovascularization

**DOI:** 10.1096/fj.202501793R

**Published:** 2025-07-26

**Authors:** Tianxi Wang, Manon Szczepan, Austin T. Gregg, Xingyan Wang, Lois E. H. Smith, Ye Sun

**Affiliations:** ^1^ Department of Ophthalmology, Harvard Medical School Boston Children's Hospital Boston Massachusetts USA

**Keywords:** choroidal neovascularization, macrophages, microglia, myeloid cells, SOCS3, Trem2

## Abstract

Choroidal neovascularization (CNV) is a hallmark of neovascular age‐related macular degeneration, a leading cause of irreversible vision loss in the elderly. While immune dysregulation and myeloid cell activation have been implicated in CNV pathogenesis, the molecular mechanisms by which myeloid subsets influence NV remain incompletely understood. Triggering receptor expressed on myeloid cells 2 (TREM2) is an immunomodulatory receptor enriched in microglia and tissue macrophages, known to play protective roles in retinal and neurodegenerative diseases. However, its function in CNV has not been fully characterized. In this study, we investigated the role of TREM2 in CNV using transcriptomic, genetic, and functional approaches. Single‐cell RNA sequencing revealed selective upregulation of *Trem2* in activated microglia and macrophages following laser‐induced CNV. These findings were validated at the protein level using immunostaining, which confirmed robust TREM2 expression in lesion‐associated IBA1^+^ myeloid cells. Functionally, *Trem2* haploinsufficiency exacerbated CNV lesion size and vascular leakage, indicating a protective role in disease modulation. Transcriptomic profiling demonstrated that *Trem2*‐expressing myeloid cells exhibit distinct angiogenic and inflammasome‐related gene signatures, suggesting that TREM2 regulates angiogenesis through modulation of inflammatory pathways. We further examined the functional interaction between TREM2 and suppressor of cytokine signaling 3 (SOCS3), another anti‐inflammatory mediator upregulated during CNV. Using compound mutant mice, we showed that Trem2 and SOCS3 function through overlapping but independent anti‐angiogenic programs, and their combined deficiency leads to additive worsening of CNV pathology. These findings establish TREM2 as a key regulator of myeloid cell function and angiogenesis in the diseased retina.

## Graphical abstract

This study demonstrates that TREM2 is selectively upregulated in activated microglia and macrophages during choroidal neovascularization (CNV). Single‐cell RNA sequencing and immunostaining confirmed its enrichment in IBA1^+^ myeloid cells near CNV lesions. Functionally, TREM2 haploinsufficiency worsened CNV lesion size and vascular leakage.

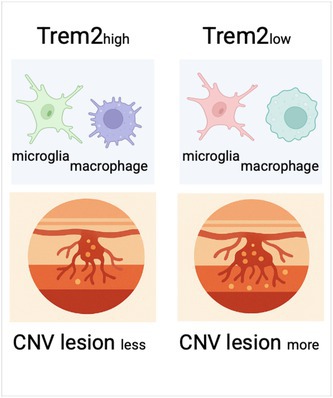

## 1 Introduction

Choroidal neovascularization (CNV) is a defining feature of neovascular age‐related macular degeneration (nAMD), a leading cause of irreversible vision loss in the elderly. CNV pathogenesis involves complex interactions among resident and infiltrating immune cells, pro‐angiogenic mediators, and tissue remodeling processes. Both clinical and experimental studies have implicated immune dysregulation in the development and progression of CNV [1, 2, 3, 4, 5, 6, 7, 8]. Myeloid immune cells have been associated with the formation of drusen, a hallmark of AMD pathology [1]. However, the molecular mechanisms through which these cells drive pathological NV remain incompletely understood.

Triggering receptor expressed on myeloid cells 2 (TREM2) is an immunomodulatory receptor predominantly expressed by resident microglia and macrophages [9]. As a transmembrane protein in the immunoglobulin superfamily, TREM2 binds to ligands such as lipids, lipoproteins, and damage‐associated molecules released during cellular stress [10, 11]. In the retina, Trem2 has been shown to play a protective role in degenerative conditions. Its expression is upregulated in human retinal degeneration, and genetic deficiency of Trem2 accelerates disease progression in mouse models by increasing microglial infiltration [12, 13]. Yu et al. recently identified a Trem2‐positive microglial subset that mitigates retinal degeneration by clearing atrophic debris in retinal degeneration mouse models [13]. Similarly, in the central nervous system, Trem2 has been shown to regulate microglial activation and phagocytosis [14, 15, 16], underscoring its neuroprotective and anti‐inflammatory functions.

Trem2 has been extensively investigated in the brain [17, 18, 19, 20], where its critical biological functions have been elucidated [14, 15, 16]. Trem2 has been implicated in lipid metabolism, inflammation suppression, and regulation of the complement cascade [9, 16, 21, 22, 23]. Loss‐of‐function mutations in Trem2 are associated with accelerated retinal neurodegeneration and increased complement activation [16]. Moreover, Trem2 deficiency exacerbates photoreceptor degeneration in models of retinal detachment [12]. However, its role in CNV pathogenesis remains unclear. Notably, a recent study demonstrated that pharmacological activation of Trem2 using the lipid agonist sulfatide can reduce pathological NV in the laser‐induced CNV model [24], further supporting its potential therapeutic relevance. Despite these findings, the mechanisms by which TREM2 regulates myeloid cell function during CNV and its broader role in retinal angiogenesis remain to be elucidated.

In this study, we investigated the role of TREM2 in CNV using an integrated approach that combined transcriptomic analysis, genetic models, and functional assays. Analysis of a publicly available single‐cell RNA sequencing (scRNA‐seq) dataset revealed selective upregulation of *Trem2* in activated myeloid subsets, particularly within microglia and macrophage clusters, following laser‐induced CNV. These transcriptomic findings were validated at the protein level through immunostaining, which confirmed robust TREM2 protein expression in CNV retinas, highlighting its dynamic regulation in vivo. Functionally, Trem2 haploinsufficiency led to significantly enlarged CNV lesions and increased vascular leakage, supporting a protective role for TREM2 in modulating CNV. Further transcriptomic profiling revealed that Trem2‐expressing microglia and macrophages exhibit distinct angiogenic and inflammasome‐associated gene signatures, suggesting that TREM2 limits pathological angiogenesis by modulating inflammatory and angiogenic signaling pathways. We also explored the relationship between TREM2 and suppressor of cytokine signaling 3 (SOCS3), another anti‐inflammatory mediator enriched in myeloid cells that becomes upregulated during CNV. In compound mutant mice, combined loss of Trem2 and SOCS3 led to additive exacerbation of CNV pathology, indicating that these factors define overlapping yet independent anti‐angiogenic programs. In summary, our findings identify TREM2 as a key regulator of myeloid cell function in CNV and provide new insights into how myeloid heterogeneity and signal diversity shape angiogenic outcomes in retinal disease.

## 2 Materials and Methods

### 2.1 Mice

All animal procedures were approved by the Institutional Animal Care and Use Committee (IACUC) at the Boston Children's Hospital and conducted following the guidance of The Association for Research in Vision and Ophthalmology (ARVO) for the ethical use of animals in vision research. C57BL/6J mice (stock#000664, RRID:IMSR_JAX:000664), LysM‐Cre mice (stock#004781, RRID:IMSR_JAX:0004781), Ai9 flox mice (stock#007909, RRID:IMSR_JAX:0007909), and *Trem2* knockout mice (stock#027197, RRID:IMSR_JAX:027197) were obtained from the Jackson Laboratory. *Socs3* flox (*Socs3* ^*f/f*^) mice [25] (a gift from Dr. A. Yoshimura) were crossed with LysM‐Cre mice to generate myeloid‐specific *Socs3* knockout mice (*Socs3* ^cKO^), which were then crossed with Trem2 knockout mice to generate compound mutants. Littermate flox/flox mice were used as control mice. Both sexes were used in all experiments.

### 2.2 Laser‐Induced CNV

CNV was induced using the Micron IV Image‐Guided Laser System (Phoenix) as previously described [26, 27]. Six‐to‐Eight‐week‐old mice were randomly divided into two groups: control group and experimental group. Mice were anesthetized with ketamine/xylazine via intraperitoneal injection, and pupils were dilated with 1% tropicamide. Laser photocoagulation was performed using the Micron IV system. Mice were euthanized at designated timepoints post‐laser, and eyes were fixed in 4% paraformaldehyde (Fisher Scientific) in 0.01 M PBS for 1 h. The retinal pigment epithelium (RPE)‐sclera‐choroid complex was dissected, permeabilized with 0.2% Triton X‐100 in PBS for 1 h, and stained with Isolectin GS‐IB4 (ThermoFisher Scientific, I21411, RRID:AB_23146) or with the indicated antibodies. After PBS washes, the RPE‐sclera‐choroid complexes were flat‐mounted using mounting medium (Vector Labs, H‐1000‐10). Images were acquired with a Zeiss AxioObserver.Z1 microscope or Zeiss 980 confocal microscope, and CNV lesions were quantified using ImageJ (RRID:SCR_003070) by researchers blinded to the subjects. Lesion exclusion criteria were based on previously published methods [26].

### 2.3 Single‐Cell RNA Sequencing Data Acquisition and Processing

The single‐cell RNA sequencing (scRNA‐seq) data was obtained from the Gene Expression Omnibus (GEO) (RRID:SCR_005012) under accession number GSE239941 [28] for ocular CD45^+^ immune cells isolated from sham and laser‐treated wildtype eyes. Barcode, feature, and matrix files in 10x Genomics format were processed using Python (RRID:SCR_024202) and the Scanpy (v1.9.3) package. The count matrix was generated using the sc.read_10x_mtx() function to load the barcode (barcodes.tsv.gz), feature (features.tsv.gz), and matrix (matrix.mtx.gz) files. Standard quality control steps were applied to remove low‐quality cells, followed by normalization and scaling. Dimensionality reduction was performed via principal component analysis (PCA), and clustering was conducted using the Leiden algorithm. Visualization was performed using Uniform Manifold Approximation and Projection (UMAP). Cluster identities were assigned based on differentially expressed genes and validated using known marker genes for microglia, macrophages, monocytes, T cells, and B cells.

### 2.4 Immunofluorescence

Mouse eyes were collected and immediately fixed in 4% paraformaldehyde at room temperature for 1 h, then washed in 1X PBS (0.01 M, pH 7.4) three times for 30 min each. The retina and RPE‐choroid complex were dissected and blocked with 2% bovine serum albumin, 5% normal goat serum, and 0.3% Triton X‐100 in PBS for 1 h at room temperature. Samples were then incubated overnight at 4°C with primary antibodies diluted in 0.3% Triton X‐100 in PBS. After washing three times with 1X PBS (0.01 M, pH 7.4) for 30 min each, tissues were incubated with fluorophore‐conjugated secondary antibodies for 1 h at room temperature in the dark, followed by three PBS washes. Stained tissues were mounted with anti‐fade medium and cover slipped. Imaging was performed using Zeiss 980 confocal microscopes. Antibodies used in this study included antibodies against: Trem2 (R&D Systems, cat#AF1729, RRID:AB_354956), IBA1 (Fujifilm, cat#011‐27 991, RRID:AB_2935833 and Synaptic Systems, cat#234009, RRID:AB_2891282), ASC (CST, cat#67824, RRID:AB_2799736), NLRP3/NALP3 (Novusbio, cat#NBP2‐12446, RRID:AB_2750946), and GSDMD (Abcam, cat#ab219800, RRID:AB_2888940). The secondary antibodies included: Goat anti‐Rabbit IgG 488 (Thermo Fisher Scientific, cat#A32731, RRID:AB_2633280), Goat anti‐Rabbit IgG 594 (Thermo Fisher Scientific, cat#A11037, RRID:AB_2534095), Goat anti‐Chicken IgG 488 (Thermo Fisher Scientific, cat#A11039, RRID:AB_2534096), Donkey Anti‐Goat IgG 488 (Thermo Fisher Scientific, cat#A‐11055, RRID:AB_2534102), Donkey Anti‐Rabbit IgG 594 (Thermo Fisher Scientific, cat#A‐21207, RRID:AB_141637), and Donkey Anti‐Rabbit IgG 647 (Thermo Fisher Scientific, cat#A‐31573, RRID:AB_2536183).

### 2.5 Quantitative PCR (qPCR)

The retina and RPE‐choroid complex were isolated under a dissecting microscope and lysed, and total RNA was extracted using an RNA extraction kit according to the manufacturer's instructions. A total of 1 μg of RNA was reverse transcribed using the iScript cDNA Synthesis Kit (Bio‐Rad, Cat# 170–8891). qPCR was performed using SYBR Green‐based detection, with *Cyclophilin A/Ppia* as the housekeeping gene for normalization. Expression levels of *Trem2* were quantified using gene‐specific primers. The primer sequences included: *Trem2* forward (5′–3′) CTG GAA CCG TCA CCA TCA CTC, *Trem2* reverse (5′–3′) CGA AAC TCG ATG ACT CCT CGG; *Ppia* forward (5′–3′) GAG CTG TTT GCA GAC AAA GTT C; and *Ppia* reverse (5′–3′) CCC TGG CAC ATG AAT CCT GG.

### 2.6 Statistics

Statistical analyses were performed using GraphPad Prism (RRID:SCR_002798). Data represent at least three independent experiments and are presented as mean ± standard error of the mean. Group sizes were determined based on prior experience with similar experimental models [27]. Unpaired nonparametric Mann–Whitney *U* Test was used for two‐group comparison. Statistical significance was defined as a *p* < 0.05.

## 3 Results

### 3.1 Trem2 Was Upregulated in Activated Myeloid Cells During CNV

**FIGURE 1:**
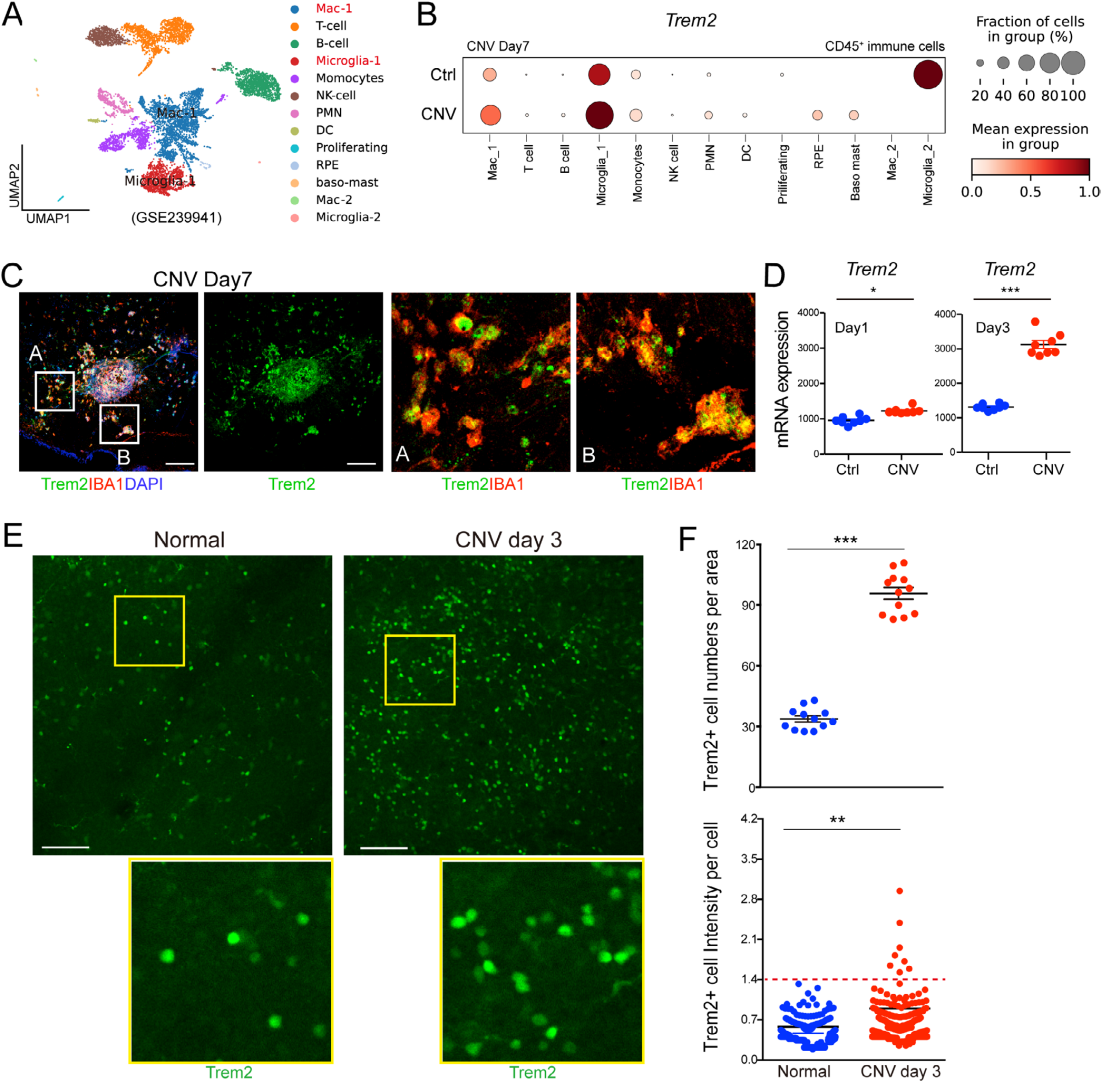
Trem2 was upregulated in myeloid cells during laser‐induced CNV. (A, B) scRNA‐seq analysis (GSE239941) revealed elevated *Trem2* expression in subsets of macrophages and microglia isolated from CNV tissues. (C) Immunostaining of retinal flat mounts from wild‐type mice showed Trem2 expression in IBA1^+^ macrophages and microglia in CNV areas. Scale bar: 100 μm. (D) Quantitative RT‐PCR analysis demonstrated increased *Trem2* mRNA expression in retinas/choroid tissues from CNV mice at day 1 and day 3 post‐laser, compared to non‐lasered controls. Ctrl: Control mice without CNV. Data are presented as mean ± SEM (*n* = 8 replicates from 4 retinas per group). (E) Representative images of retinal flat mounts facing RPE side from CNV mice showed enhanced Trem2 staining compared to non‐lasered controls. Scale bar: 100 μm. (F) Quantification of Trem2^+^ cell number per area (*n* = 12 retinal regions per group) and Trem2 staining intensity per Trem2^+^ cell confirmed significant upregulation of Trem2 expression in the CNV model. *p < 0.05; **p < 0.01; ***p < 0.001.

To investigate the role of Trem2 in vascular eye diseases, we analyzed a published scRNA‐seq dataset (GSE239941) of CD45^+^ immune cells from retinal and choroidal tissues after laser‐induced CNV (Figure 1A,B). *Trem2* expression was predominately upregulated in specific immune cell subsets, particularly in the microglia_1 and macrophage_1 (Mac_1) clusters. *Trem2* is highly elevated in the microglia_2 cluster in the control group, but this subset contained very few cells and is unlikely to significantly contribute to CNV development. This selective upregulation suggests a potential role of Trem2 in modulating immune responses during CNV pathogenesis. Immunostaining of retinal tissues at day 7 after post‐induced confirmed strong Trem2 expression in IBA1^+^ myeloid cells accumulated at the sites of laser injury (Figure 1C). This data confirms that Trem2 expression is localized to activated myeloid cells, particularly microglia and macrophages, in the CNV lesion areas. qPCR analysis showed that *Trem2* mRNA levels were significantly increased as early as day 1 and continued rising through day 3 post‐laser (Figure 1D), indicating early and sustained transcriptional activation. We also observed that the Trem2^+^ cell population was significantly increased by approximately threefold in the CNV retinas compared to normal controls (Figure 1E,F). Additionally, increased Trem2 staining intensity was detected in only about 8% of the Trem2^+^ cells, suggesting a potential shift in cell population rather than uniform upregulation of Trem2 during CNV. Together, these findings highlight Trem2 as a dynamically regulated gene in activated myeloid cells during CNV, implicating its involvement in immune‐mediated pathological angiogenesis in the retina and choroid.

### 3.2 Trem2 Haploinsufficiency Promoted CNV and Vascular Leakage

**FIGURE 2:**
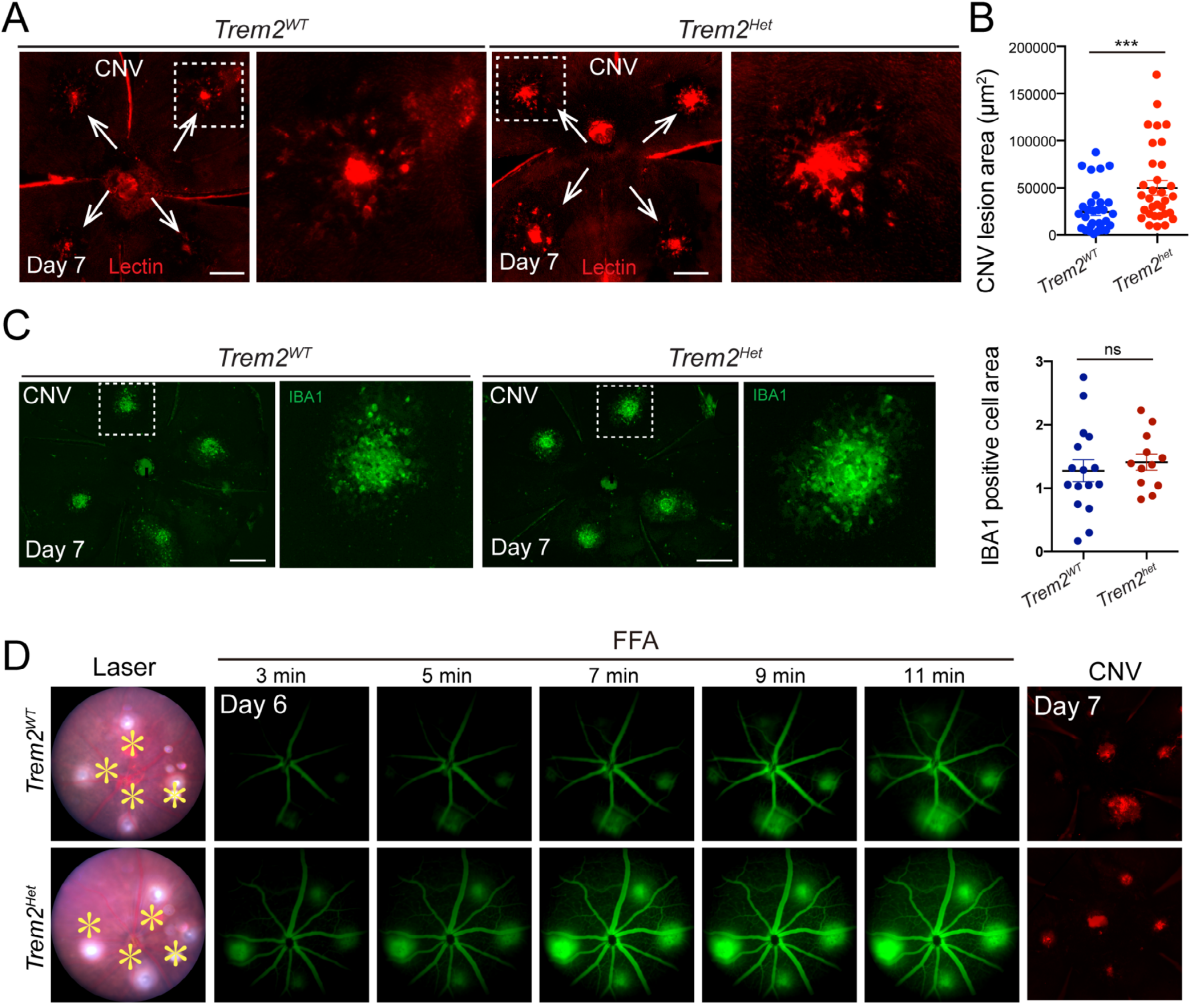
Trem2 haploinsufficiency enhanced CNV and vascular leakage. (A) Representative images of CNV lesions in choroidal flat mounts from Trem2 wild‐type (*Trem2* ^*+/+*^) and Trem2 heterozygous (*Trem2* ^*+/−*^) mice at day 7 post‐laser, stained with Lectin (red). Scale bar: 500 μm. (B) Quantification of CNV lesion area revealed significantly larger lesions in *Trem2* ^*+/−*^ mice (*n* = 30 lesions) compared to *Trem2* ^*+/+*^ controls (*n* = 24 lesions). Data are presented as mean ± SEM. ***p < 0.001. (C) Immunostaining for IBA1 and quantification showed myeloid cell population in CNV lesions in *Trem2* ^*+/−*^ mice (*n* = 12). Scale bar: 500 μm. (D) Representative fundus fluorescein angiography (FFA) demonstrated greater vascular leakage in *Trem2* ^*+/−*^ mice relative to wild‐type controls (*n* = 6 eyes per group).

Trem2 haploinsufficiency has been linked to an increased risk for Alzheimer's disease and microglial dysfunction [16], while complete TREM2 loss causes Nasu‐Hakola disease [29], an autosomal recessive genetic disorder that affects both the central nervous system and the skeleton. To study how TREM2 contributes to nAMD pathogenesis, we used Trem2 haploinsufficiency mice (*Trem2* ^*+/−*^). Compared to Trem2 wild‐type (*Trem2* ^*+/+*^) littermates, *Trem2* ^*+/−*^ mice exhibited significantly larger CNV lesions (Figure 2A,B), despite comparable amounts of IBA1^+^ myeloid cells within the CNV areas (Figure 2C), suggesting a protective role for Trem2 in limiting pathological CNV. This was supported by more pronounced vascular leakage observed on fundus fluorescein angiography (Figure 2D), indicating impaired vascular integrity. Together, these findings suggest that Trem2 haploinsufficiency regulates angiogenic responses, contributing to CNV. Thus, Trem2 acts as a negative regulator of pathological angiogenesis and may help maintain retinal homeostasis during disease.

### 3.3 Trem2‐Expressing Microglia and Macrophages Show Pro‐Angiogenic and Inflammasome‐Related Gene Signatures in CNV

**FIGURE 3:**
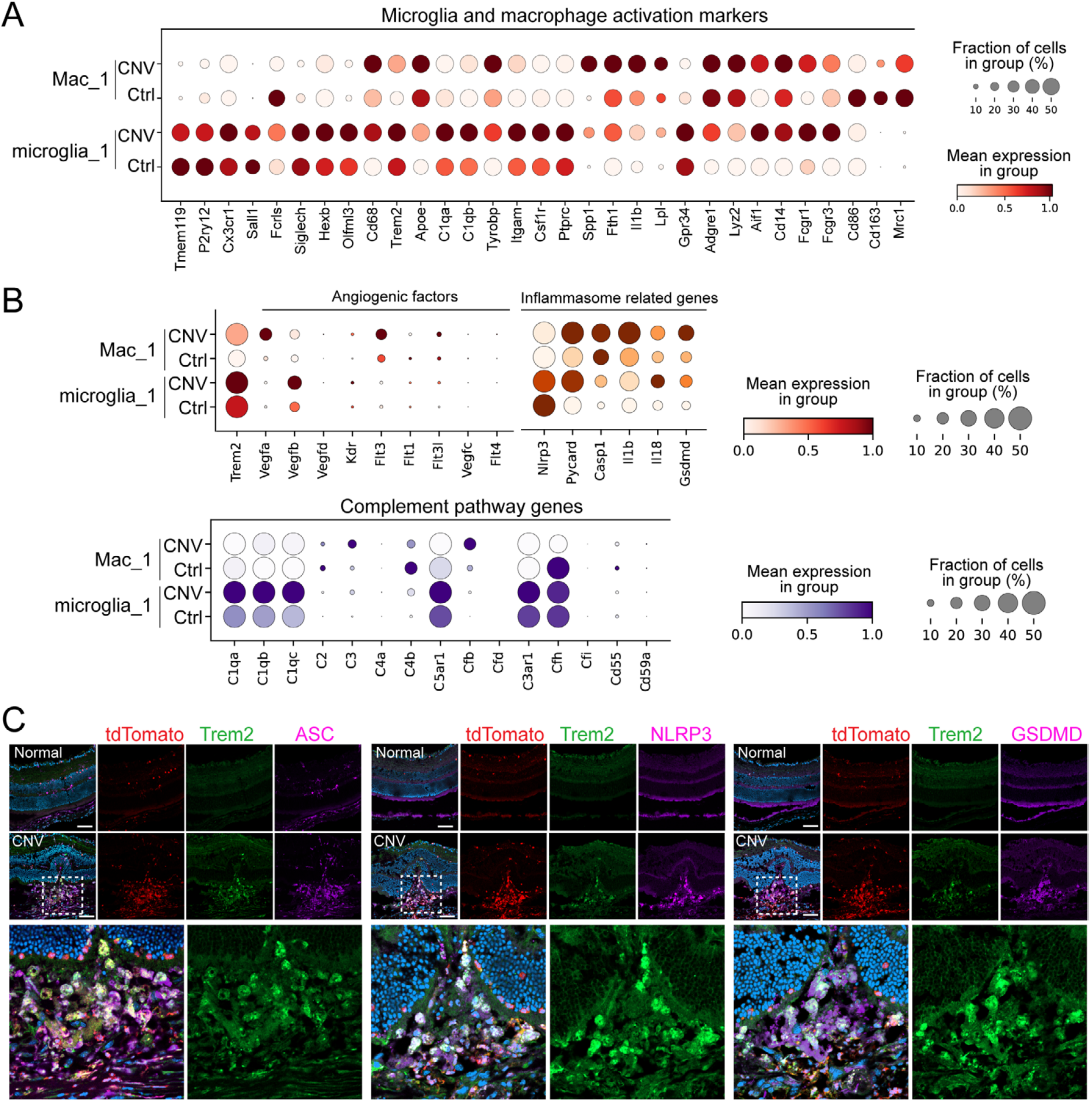
Trem2‐expressing microglia and macrophages exhibit pro‐angiogenic, inflammasome‐related, and complement‐associated gene signatures in CNV. (A) Single‐cell RNA sequencing analysis of CD45^+^ immune cells from control and laser‐induced CNV tissues identified distinct gene expression profiles in microglia_1 and macrophage (Mac_1) clusters. (B) scRNA‐seq analysis showing the expression of angiogenesis‐ and inflammasome‐associated genes as well as complement pathway genes in microglia_1 and Mac_1 under normal and CNV conditions. (C) Immunofluorescence staining confirmed increased protein expression of ASC, NLRP3, and GSDMD in tdTomato^+^ myeloid cells in CNV lesions from LysM‐Cre driven Ai9 tdTomat reporter mice (*n* = 4 retinas/group). Scale bar: 200 μm.

To elucidate how Trem2 contributes to angiogenesis, we performed in‐depth transcriptomic analysis of immune cells from laser‐induced CNV lesions (GSE239941). We focused on the microglia_1 and (Mac_1) clusters, where Trem2 is upregulated (Figure 1A), and assessed their transcriptional profiles. To better define the identity and activation status of microglia and macrophages in CNV, we examined the expression of canonical marker genes (Figure 3A). In the microglia_1 cluster, CNV samples showed reduced expression of key homeostatic microglial markers *Tmem119*, *P2ry12*, and *Sall1*, suggesting a loss of microglial homeostasis in the lesioned areas. In contrast, other microglia‐enriched genes such as *Cx3cr1*, *Fcrls*, *Siglech*, *Hexb*, and *Olfml3* were elevated, indicating enhanced microglial presence or proliferation within the lesioned areas. Concurrently, there was robust upregulation of activation‐ and disease‐associated microglial genes including *Cd68*, *Trem2*, *Apoe*, *C1qa*, *C1qb*, *Tyrobp*, *Itgam*, *Csf1r*, *Ptprc*, *Spp1*, *Fth1*, *Il1b*, *Lpl*, and *Gpr34*, reflecting a shift toward a metabolically active and pro‐inflammatory phenotype. These transcriptional changes suggest that microglia in CNV lesions undergo metabolic reprogramming—marked by increased glycolysis, lipid uptake, and iron handling—alongside enhanced phagocytic and inflammatory activity. Overall, the gene expression profile indicates a transition from a homeostatic to an activated microglial state, consistent with responses to injury and inflammatory stimuli in CNV pathology. Notably, the enrichment of *Trem2* and its downstream effectors (*Apoe*, *Tyrobp*, and *Lpl*) [30, 31] points to a population of Trem2^+^ microglia engaged in lipid processing, phagocytosis, and immunomodulation, potentially playing a key role in orchestrating both inflammatory and reparative responses within the CNV microenvironment.

In the Mac_1 cluster, we observed increased expression of genes associated with phagocytosis and immune activation (*Cd68, Cd14, Fcgr1, Fcgr3, Aif1*, and *Lyz2*), lipid metabolism and stress responses (*Apoe, Lpl, Spp1, Fth1*, and *Tyrobp*), and maintenance of resident identity (*Adgre1*). Concurrently, there was downregulation of anti‐inflammatory and reparative markers (*Cd86, Cd163*, and *Mrc1*). This gene signature reflects a loss of anti‐inflammatory or tissue‐resolving functions, indicative of a transition toward a disease‐associated macrophage‐like phenotype. These results suggest that macrophages in CNV undergo a functional shift from a homeostatic, tissue‐repairing state toward a pro‐inflammatory, lipid‐laden, and potentially pro‐angiogenic phenotype, contributing to chronic inflammation, lipid dysregulation, and pathological tissue remodeling.

Given the strong link between inflammation and NV, we next analyzed angiogenesis‐ and inflammasome‐related genes across both populations (Figure 3B). Notably, *Vegfa* and *Il1b*, key drivers of angiogenesis and inflammation, were preferentially upregulated in Mac_1, whereas *Vegfb* and *Il18* were more selectively elevated in microglia_1 in CNV samples. This suggests that while both cell types contribute to pathological angiogenesis, they may do so via distinct molecular pathways. We also observed upregulation of *Pycard* and *Gsdmd*, essential components of the inflammasome complex, in both microglia_1 and Mac_1, indicating active inflammasome signaling in CNV lesions. Complement pathway genes, which are often downstream of inflammasome activation and known to promote NV, were also highly expressed and upregulated in microglia, supporting a central role for microglial complement activation in CNV pathology. To confirm these transcriptomic findings at the protein level, we performed immunostaining for key inflammasome‐related markers, including ASC (encoded by *Pycard*), NLRP3, and GSDMD, in retinal tissues with or without CNV. Immunofluorescence revealed strongly upregulated levels of these proteins in IBA1^+^ macrophages and microglia within CNV lesions, supporting the scRNA‐seq data and suggesting that inflammasome activation is a shared molecular signature of myeloid cells in CNV lesions (Figure 3C).

Together, these findings suggest that myeloid Trem2 may regulate angiogenesis, in part, through modulation of inflammasome activity, complement pathway activation, and angiogenic factors expression, highlighting its protective role in CNV pathogenesis.

### 3.4 Myeloid Trem2 and SOCS3 Define Distinct and Overlapping Anti‐Angiogenic Programs During CNV

**FIGURE 4:**
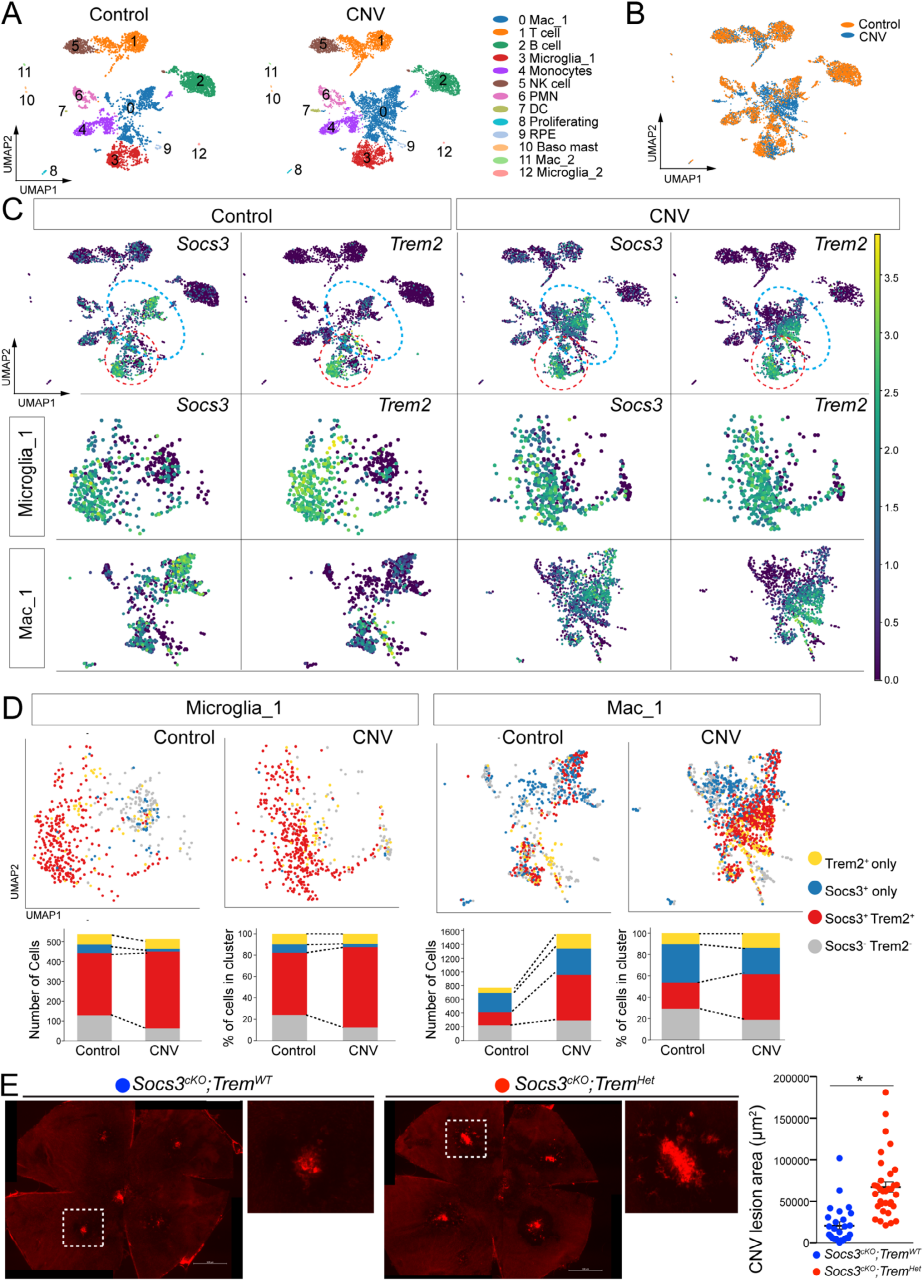
Myeloid Trem2 and SOCS3 define distinct and overlapping anti‐angiogenic programs in CNV. (A) UMAP visualization of single‐cell RNA‐seq data (GSE239941) showing 13 distinct immune cell populations identified in retinal and choroidal tissues, annotated by canonical marker gene expression. (B) Distribution of immune cell clusters in no‐laser control and laser‐induced CNV conditions, highlighting shifts in immune landscape upon CNV induction. (C) Expression patterns of *Trem2* and *Socs3* across myeloid compartments in CNV compared to controls. (D) Proportional analysis of Trem2^+^, Socs3^+^, double‐positive (Trem2^+^Socs3^+^), and double‐negative (Trem2^‐^Socs3^‐^) populations within myeloid cells reveals a marked expansion of Trem2^+^Socs3^+^ macrophages in CNV. (E) Quantification of CNV lesion areas in myeloid‐specific *Socs3* conditional knockout (cKO) mice (*n* = 22 lesions) and *Socs3* cKO;*Trem2* ^*+/−*^ compound mutants (*n* = 30 lesions) demonstrates that *Trem2* haploinsufficiency further exacerbates CNV. *p < 0.05. Scale bar: 500 μm.

SOCS3 is another anti‐angiogenic factor, and our previous studies demonstrated its protective role in CNV [27]. To better understand the cellular landscape and roles of myeloid Trem2 and its interaction with Socs3 in CNV, we analyzed dataset (GSE239941) and identified 13 distinct immune cell populations (Figure 4A), annotated based on canonical marker gene expression. The distribution of these clusters differed substantially between no‐laser controls and laser‐induced CNV samples (Figure 4B), reflecting robust immune cell recruitment and activation in response to CNV and emphasizing the functional plasticity and heterogeneity of myeloid cells in disease progression.

Given our earlier observations of Trem2 upregulation in myeloid cells during CNV and the known anti‐inflammatory and neuroprotective roles of SOCS3, we examined their expression patterns across myeloid compartments (Figure 4C). Both Trem2 and Socs3 were significantly elevated in CNV conditions. To explore their relationship, we stratified myeloid cells into Trem2^+^, Socs3^+^, double‐positive, and double‐negative subsets. Notably, Trem2^+^Socs3^+^ double‐positive cells were significantly expanded in the CNV group, with the most pronounced increase observed in the Mac_1 cluster (Figure 4D). The Microglia_1 cluster also showed a moderate increase in double‐positive cells. This shift from Trem2/Socs3 double‐negative or single‐positive cells to double‐positive cells was more prominent in the Mac_1 cluster, suggesting that macrophage‐specific co‐expression of Trem2 and Socs3 may play a dominant role in modulating CNV pathology. The presence of single‐positive and double‐negative subsets further indicates that Trem2 and Socs3 may also function independently, contributing to distinct and potentially non‐overlapping roles during CNV development.

To assess functional interactions, we generated Socs3 conditional knockout (cKO) mice using LysM‐Cre mice, enabling myeloid‐specific deletion. Myeloid Socs3 deficiency exacerbated CNV [27], reinforcing its protective role in retinal inflammation and NV. We next crossed *Socs3* ^*cKO*^ mice with Trem2 heterozygous (*Trem2* ^+/−^) mice to generate *Socs3* ^*cKO*^;*Trem2* ^+/−^ compound mutants. Trem2 haploinsufficiency significantly further aggravated CNV lesion size (Figure 4E), suggesting that Trem2 and Socs3 function through distinct yet additive mechanisms to suppress pathological angiogenesis. These results highlight the independent and cooperative roles of Trem2 and Socs3 in myeloid cells during CNV and underscore the importance of myeloid heterogeneity and molecular crosstalk in regulating retinal angiogenesis.

## 4 Discussion

In this study, we identified Trem2 as a key immunoregulatory gene in retinal myeloid cells during CNV, highlighting its protective role against pathological angiogenesis in a model of nAMD. Using single‐cell transcriptomics, genetic perturbation, and immunohistochemistry, we demonstrated that Trem2 is dynamically upregulated in activated macrophages and microglia following laser‐induced CNV, and that *Trem2* haploinsufficiency exacerbates CNV severity. Our findings align with recent studies implicating Trem2 in the regulation of immune response in AMD [24]. Yagi et al. showed that pharmacologic activation of Trem2 signaling via sulfatide reduces CNV lesion size and local inflammation [24], consistent with our observation that *Trem2* ^*+/−*^ mice develop larger, more leaky lesions. These complementary results support the conclusion that Trem2 is not merely a marker of immune activation but an active suppressor of CNV pathogenesis. Interestingly, while Yagi et al. reported no significant CNV phenotype change in *Trem2* knockout mice compared to C57BL6 mice [24], our findings and previous studies [16] suggest that Trem2 haploinsufficiency may increase vulnerability to pathological angiogenesis and neurodegeneration. This discrepancy may be attributed to the role of Trem2 in regulating complement activation [16]. Trem2 binds to C1q, a key component of the classical complement pathway, thereby inhibiting complement activation under physiological conditions. However, Trem2 haploinsufficiency may lead to dysregulated complement activation, increased C3 expression, and heightened inflammation, ultimately promoting neurodegeneration and vascular dysfunction in mouse models.

Additionally, complete loss of Trem2 from early development may trigger compensatory upregulation of alternative pathways (e.g., other TAM receptors, DAP12‐independent signaling) that mask the phenotype. In contrast, Trem2 haploinsufficiency retains partial Trem2 function, possibly avoiding full compensation and revealing a sensitized disease state where dysfunction becomes evident under stress (e.g., CNV). Studies in the brain [18] have shown that Trem2^−/−^ microglia fail to adopt the disease‐associated microglia phenotype, indicating that complete loss of Trem2 leads to distinct transcriptional states that may be incompatible with supporting angiogenic or inflammatory responses in CNV conditions.

Furthermore, our findings align with a recent study [13] that identified TREM2‐expressing microglia as a distinct population localized near drusen in human AMD tissues. That study identified a specific subset of TREM2^+^ and galectin‐3 (GAL3^+^) microglia enriched in the macular region of patients with AMD, particularly in the subretinal space and in areas of geographic atrophy. The frequency of these cells correlated positively with AMD severity. Flow cytometry further confirmed an increased number of TREM2^+^ myeloid cells in the RPE/choroid tissues of AMD donors, supporting a conserved role for TREM2‐expressing microglia in human AMD pathogenesis. Consistently, our work demonstrated that Trem2^+^ macrophages and microglia in acute injury similarly upregulate lipid‐handling and complement genes (*Apoe*, *C1qa*, and *Tyrobp*), suggesting a conserved transcriptional program of tissue protection across chronic and acute retinal stress.

Crucially, our transcriptomic analysis revealed that *Trem2* is enriched in microglia and macrophage subsets co‐expressing genes associated with angiogenesis (*Vegfa* and *Vegfb*), inflammasome signaling (*Il1b*, *Il18*, *Pycard*, and *Gsdmd*), and complement activation. These findings suggest that *Trem2* not only influences the magnitude of immune responses during CNV but also shapes their quality, balancing between tissue repair with the suppression of destructive inflammation. The expression of inflammasome‐related transcripts and proteins in *Trem2* ^+^ cells implicates a mechanism in which *Trem2* may constrain excessive inflammatory signaling that drives NV.

Our single‐cell analysis further highlighted the heterogeneity of CD45^+^ immune cells following laser CNV. We observed a notable increase in macrophage population, while microglial population remains relatively stable. Notably, both Trem2 and Socs3 were significantly upregulated in CNV. We also observed a significant increase in Trem2^+^Socs3^+^ double‐positive cells, particularly in macrophages, indicating this subset may play a central role in regulating angiogenesis and inflammation in response to CNV. The presence of Trem2 or Socs3 single‐positive and double‐negative populations further highlights the functional heterogeneity of myeloid cells and suggests that distinct transcriptional programs may underlie diverse roles in CNV.

SOCS3 is a well‐established immunoregulatory molecule that limits inflammation by inhibiting STAT3 signaling. Our previous research [27] demonstrated that SOCS3 acts as a protective factor in laser‐induced CNV by inhibiting excessive inflammation and maintaining tissue homeostasis. To further explore the interplay between SOCS3 and Trem2 in regulating myeloid cell function during CNV, we generated myeloid Socs3‐deficient mice with Trem2 haploinsufficiency (*Trem2* ^*+/−*^) compound mutants. Even in the absence of SOCS3, Trem2 deficiency further exacerbated CNV severity. The expansion of Trem2^+^SOCS3^+^ macrophages in CNV lesions suggests the presence of a regulatory axis within the myeloid compartment that buffers against angiogenic and inflammatory signaling. While SOCS3 deletion alone increased CNV lesion size, this effect was further amplified by Trem2 haploinsufficiency, supporting the concept that targeting multiple immune checkpoints may be required to fully suppress maladaptive myeloid responses. Further studies are warranted to determine how SOCS3 and Trem2 cooperatively regulate inflammation and angiogenesis in CNV, which could lead to the identification of novel therapeutic targets for mitigating pathological NV.

Our study advances the field in several key ways. First, it demonstrates that Trem2 is not merely associated with AMD pathology but actively confers protective effects in vivo. Second, it uncovers transcriptomic signatures that suggest mechanistic pathways—complement, inflammasome, VEGF signaling—through which Trem2^+^ cells mediate these effects. Third, it establishes that Trem2 functions in parallel with SOCS3, revealing new molecular diversity in myeloid regulatory programs. These insights provide a framework for rethinking how innate immunity contributes to CNV—not solely as a driver of disease but also as a potential source of endogenous regulation and resolution. Clinically, these findings are highly relevant. While anti‐VEGF therapies are the standard of care, they fail to fully control disease in a substantial subset of patients, and do not address the underlying inflammatory drivers of NV [32]. Our results, in concert with other studies, suggest that pharmacologic activation of TREM2 or enhancement of its downstream pathways may offer a new strategy to stabilize the immune microenvironment in nAMD. Such immune‐modulating approaches could serve as additive or synergistic treatments alongside anti‐angiogenic therapy. In conclusion, we define Trem2 as a critical negative regulator of pathological NV, acting through transcriptional and functional modulation of macrophages and microglia. These findings position Trem2 at the intersection of lipid sensing, inflammation, and angiogenesis, and establish it as a promising therapeutic target in AMD and other neovascular retinopathies.

## Author Contributions

T.W. and Y.S. designed experiments; T.W., M.S., A.T.G., and X.W. conducted experiments; T.W. and Y.S. analyzed the data; T.W., L.S., and Y.S. wrote the manuscript; all the authors reviewed and approved the manuscript.

## Conflicts of Interest

Y.S., L.S., and T.W. are inventors on patent applications filed by Boston Children's Hospital. The remaining authors declare no conflicts of interest.

## Data Availability

The data that support the findings of this study are available from the corresponding authors upon reasonable request. The scRNA‐seq data used in this study are openly available in the Gene Expression Omnibus (GEO) (accession number GSE239941).
